# Genetic Profiling of the Isoprenoid and Sterol Biosynthesis Pathway Genes of *Trypanosoma cruzi*

**DOI:** 10.1371/journal.pone.0096762

**Published:** 2014-05-14

**Authors:** Raúl O. Cosentino, Fernán Agüero

**Affiliations:** Instituto de Investigaciones Biotecnológicas – Instituto Tecnológico de Chascomús, Universidad de San Martín – CONICET, Sede San Martín, Buenos Aires, Argentina; Federal University of São Paulo, Brazil

## Abstract

In *Trypanosoma cruzi* the isoprenoid and sterol biosynthesis pathways are validated targets for chemotherapeutic intervention. In this work we present a study of the genetic diversity observed in genes from these pathways. Using a number of bioinformatic strategies, we first identified genes that were missing and/or were truncated in the *T. cruzi* genome. Based on this analysis we obtained the complete sequence of the ortholog of the yeast ERG26 gene and identified a non-orthologous homolog of the yeast ERG25 gene (sterol methyl oxidase, SMO), and we propose that the orthologs of ERG25 have been lost in trypanosomes (but not in Leishmanias). Next, starting from a set of 16 *T. cruzi* strains representative of all extant evolutionary lineages, we amplified and sequenced ∼24 Kbp from 22 genes, identifying a total of 975 SNPs or fixed differences, of which 28% represent non-synonymous changes. We observed genes with a density of substitutions ranging from those close to the average (∼2.5/100 bp) to some showing a high number of changes (11.4/100 bp, for the putative lathosterol oxidase gene). All the genes of the pathway are under apparent purifying selection, but genes coding for the sterol C14-demethylase, the HMG-CoA synthase, and the HMG-CoA reductase have the lowest density of missense SNPs in the panel. Other genes (TcPMK, TcSMO-like) have a relatively high density of non-synonymous SNPs (2.5 and 1.9 every 100 bp, respectively). However, none of the non-synonymous changes identified affect a catalytic or ligand binding site residue. A comparative analysis of the corresponding genes from African trypanosomes and *Leishmania* shows similar levels of apparent selection for each gene. This information will be essential for future drug development studies focused on this pathway.

## Introduction

*Trypanosoma cruzi*, a protozoan parasite of the order Kinetoplastida, is the causative agent of Chagas Disease, a neglected disease that is endemic in South America, affecting in excess of 8 million people [1]. The currently available drugs used to treat Chagas Disease (Nifurtimox, Benznidazole) have several drawbacks including toxicity, and the fact that they are mostly effective during the acute phase of the infection.

The *T. cruzi* species has a structured population, with a predominantly clonal mode of reproduction, with infrequent genetic exchange [2], [3]. Through the use of a number of genetic markers the population has been divided into six evolutionary lineages, also called Discrete Typing Units (DTUs) [4], [5], now designated as TcI to TcVI [6]. Lineages TcV and TcVI (this latter lineage includes the strain used for the first genomic sequence of *T. cruzi*, CL Brener) have a very high degree of heterozygosis. The currently favoured hypothesis suggests that these two lineages originated after one or two ancestral hybridization events [7]–[9]. There are no general agreement regarding the estimated time of divergence of the six *T. cruzi* lineages; however recent estimations suggest that major lineages (excluding the hybrid lineages TcV and TcVI) diverged 1–4 millions of years ago [9], [10] while the hybrid lineages emerged much more recently (less than 1 million years ago, according to Flores-Lopez and Machado [9], and within the last 60,000 years, according to Lewis MD, *et al.* [10]). This divergence was accompanied with a diversification of phenotypic and biological properties. Indeed, several investigations suggest that the observed diversity in host preference, cell tropism and drug susceptibility might be properties of different strains and/or lineages [11]–[14].

The parasite ergosterol biosynthesis pathway is one of the major routes for chemotherapeutic intervention against *T. cruzi*. Humans and trypanosomes share many of the enzymes leading to essential isoprenoid and sterol precursors. However, the mevalonate pathway is more related to that found in bacteria and archaea [15], and the ergosterol pathway is essentially similar to that in fungi, with a number of key steps that differ with the cholesterol biosynthesis pathway of mammals.

Inhibitors that block sterol biosynthesis or the biosynthesis of isoprenoid precursors inhibit growth of the parasite and cause severe morphological defects [16], [17]. Triazole derivatives that inhibit the parasite C14-*α* sterol demethylase are the most promising compounds, with proved curative activity in murine models of acute and chronic Chagas disease [18]–[22]. And one of them (posaconazole) is undergoing a number of clinical trials. Other ergosterol biosynthesis inhibitors with good potency *in vitro* or *in vivo*, or with good activity in an enzymatic assasy include those that target 3-hydroxy-3-methyl-glutaryl-CoA reductase [23]–[25], farnesyl diphosphate syntetase [26], squalene synthase[27], [28], squalene epoxidase/monooxygenase [29], [30], lanosterol synthase/oxidosqualene cyclase [31]–[33], and 24-C sterol methyl transferase [34]–[36], as well as compounds with dual mechanisms of action (ergosterol biosynthesis inhibition and free radical generation, reviewed in [16], [37]).

The azoles, like the triazoles, are used extensively for the treatment of fungal infections with excellent results, though different resistant strains have appeared over time in different species. One of the main resistance mechanisms observed is based on a diminished affinity of the target enzyme for the compound; which is caused by specific mutations in the ERG11/CYP51 gene. Different point mutations were identified in several fungi species as responsible for this azole resistance (reviewed in [38], [39]).

In this paper we analyze the genetic diversity present in the ergosterol biosynthesis pathway of *T. cruzi* and describe the apparent genetic selective pressure on these genes.

## Results

### Filling pathway holes: genes involved in sterol biosynthesis in *T. cruzi*

To analyze the genetic diversity of the *T. cruzi* sterol biosynthesis pathway (SBP) we decided to sequence all enzymes of the pathway, starting from enzymes that produce the terpenoid backbone precursors, and going down to the last enzyme that produces ergosterol as a product. Therefore, as a first step we looked for *T. cruzi* genes that were mapped to the corresponding KEGG metabolic pathway maps [40]. SBP genes in KEGG are classified in two maps: the steroid biosynthesis pathway map (TCR00100, www.genome.jp/kegg/pathway/tcr/tcr00100.html, and the terpenoid backbone biosynthesis pathway map (TCR00900, www.genome.jp/kegg/pathway/tcr/tcr00900.html). These maps contain information derived from the *T. cruzi* CL-Brener reference genome. From this analysis we were able to identify 15 genes mapped to these pathways. However, we also detected a number of holes in the pathway: enzymatic reactions with no enzyme mapped, and cases in which the enzymes available in KEGG were truncated (probably because of genome assembly problems). Therefore, before attempting to amplify and sequence the corresponding genes, we invested some effort in analyzing the existing sequence data to obtain a relevant complement of genes. As mentioned, in one case the corresponding genes from the reference genome were truncated, probably because of genome assembly problems. This was the case of the isopentenyl-diphosphate delta-isomerase gene (TcIDI1), which was cloned and sequenced by Dr. TK Smith (unpublished, GenBank accession number: AJ866772, 1071 bp). The corresponding genes in KEGG, mapped from the CL-Brener genome (Esmeraldo-like and non-Esmeraldo-like alleles) were both shorter, at 537 bp (TcCLB.408799.19), and 540 bp (TcCLB.510431.10). Therefore for this work we used the full-length TcIDI1 sequence obtained from GenBank (AJ866772). This sequence produces an active enzyme when expressed in an heterologous system (Dr. TK Smith, personal communication).

To fill in other identified gaps, we used the *Saccharomyces cerevisiae* sterol biosynthetic pathway as a reference model (Table 1). The yeast SBP has been studied extensively, and is essentially complete in pathway databases. Using the yeast genes from these pathways, we looked for orthologs in *T. cruzi* by doing sequence similarity searches (BLAST) against the complete *T. cruzi* genome or using databases of orthologs compiled from complete genome data, such as the OrthoMCL database [41]. As a result of this strategy, we were able to map five additional genes (see Table 2), which are the orthologs of the *S. cerevisiae* genes: ERG13 (3-hydroxy-3-methylglutaryl-CoA (HMG-CoA) synthase), ERG7 (lanosterol synthase), ERG26 (C-3 sterol dehydrogenase), ERG3, (C-5 sterol desaturase), and ERG5 (C-22 sterol desaturase). These genes were present in the *T. cruzi* genome, but were not mapped to the corresponding metabolic maps in KEGG. Some of these enzymes were already characterized biochemically in *T. cruzi* [25], [32]. In all these cases except for one (ERG26), the identification of the corresponding ortholog did not present further complications. The putative *T. cruzi* ortholog of the yeast ERG26 gene (TcCLB.510873.10, C3-sterol dehydrogenase) was found in the *T. cruzi* genome database as a truncated gene of 675 bp (224 aa). This may explain why it was not mapped to the corresponding pathway map in KEGG. Reasoning that this might be a consequence of assembly problems, as in the case of the TcIDI1 gene, we set out to identify the missing portions of this gene by performing BLASTN searches against a database of unassembled genomic reads (GSS or WGS reads) from the CL-Brener genome project (data provided originally by the TIGR-SBRI-KI sequencing consortium). Starting with the truncated TcCLB.510873.10 sequence as query, we identified a number of matching sequence reads (BLASTN, E-value < 10*E* ^−40^). These sequences were then assembled into a single contiguous sequence, which was used as a query in successive rounds of BLASTN searches, followed by reassembly. This iteration cycle was repeated until we recovered the complete (full-length) sequence of the *T. cruzi* putative C3-sterol dehydrogenase gene, as judged by its alignment against the yeast ERG26 gene. This reconstructed full-length sequence of the *T. cruzi* C-3 sterol dehydrogenase gene has 1,221 bp and has been used to design primers for amplification from *T. cruzi* DNA (see Methods). The final sequence, obtained after amplification and sequencing from CL-Brener DNA, was submitted to GenBank under the accession number JN050853.

**Table 1:** Genes in the sterol biosynthesis pathway (SBP) of yeast.

Gene ID/Locus	Molecular Function	EC Number	Pathway Order	Ortholog Group
ERG10/YPL028W	Acetoacetyl-CoA thiolase	2.3.1.9	1	OG4_10214
ERG13/YML126C	3-hydroxy-3-methylglutaryl-CoA (HMG-CoA) synthase	2.3.3.10	2	OG4_11016
HMG1/2/YML075C, YLR450W	HMG-CoA reductase	1.1.1.34	3	OG4_11458
ERG12/YMR208W	Mevalonate kinase	2.7.1.36	4	OG4_11698
ERG8/YMR220W	Phosphomevalonate kinase	2.7.4.2	5	OG4_15366
ERG19/YNR043W	Mevalonate pyrophosphate decarboxylase	4.1.1.33	6	OG4_11688
ERG20/YJL167W	Farnesyl pyrophosphate synthetase	2.5.1.10	7	OG4_11009
IDI1/YPL117C	Isopentenyl diphosphate isomerase (IPP isomerase)	5.3.3.2	7'	OG4_12197
ERG9/YHR190W	Farnesyl-diphosphate farnesyl transferase (squalene synthase)	2.5.1.21	8	OG4_13084
ERG1/YGR175C	Squalene epoxidase	1.14.99.7	9	OG4_13490
ERG7/YHR072W	Lanosterol synthase (oxidosqualene cyclase)	5.4.99.7	10	OG4_11381
ERG11/YHR007C	Lanosterol 14-alpha-demethylase	1.14.13.70	11	OG4_12975
ERG24/YNL280C	C-14 sterol reductase	1.3.1.70	12	OG4_12018
ERG25/YGR060W	C-4 methyl sterol oxidase	1.14.13.72	13	OG4_13007
ERG26/YGL001C	C-3 sterol dehydrogenase	1.1.1.170	14	OG4_12533
ERG27/YLR100W	3-keto sterol reductase	1.1.1.270	15	OG4_16167
ERG6/YML008C	Delta(24)-sterol C-methyltransferase	2.1.1.41	16	OG4_13307
ERG2/YMR202W	C-8 sterol isomerase	5.-.-.-	17	OG4_14573
ERG3/YLR056W	C-5 sterol desaturase (lathosterol oxidase)	1.3.3.-	18	OG4_12421
ERG5/YMR015C	C-22 sterol desaturase	1.14.14.-	19	OG4_14688
ERG4/YGL012W	C-24(28) sterol reductase	1.3.1.71	20	OG4_16908

The table lists the reference genes used in this work for mapping the corresponding *T. cruzi* genes. Information for this table was derived from the Saccharomyces Genome Database (SGD, yeastgenome.org). Ortholog group identifiers are from the OrthoMCL Database, version 4 (orthomcl.org).

**Table 2:** Genes in the *Trypanosoma cruzi* sterol biosynthesis pathway.

SBP Ortholog	T. cruzi Locus Identifiers(s)	Current annotation	Ortholog Group	In KEGG?	Gene Name
ERG10	TcCLB.511003.60	Hypothetical protein	OG4_10214	Yes	TcACAT
ERG13	TcCLB.511903.40, TcCLB.511071.50	Hypothetical protein, conserved	OG4_11016	No	TcHMGS
HMG1, HMG2	TcCLB.509167.20, TcCLB.506831.40	3-hydroxy-3-methylglutaryl-CoA reductase, putative	OG4_11458	Yes	TcHMGR
ERG12	TcCLB.436521.9, TcCLB.509237.10	Mevalonate kinase, putative	OG4_11698	Yes	TcMK
ERG8	TcCLB.508277.140, TcCLB.507913.20	Phosphomevalonate kinase e-like protein, putative	OG4_15366	Yes	TcPMK
ERG19	TcCLB.507993.330, TcCLB.511281.40	Diphosphomevalonate decarboxylase, putative	OG4_11688	Yes	TcMVD
ERG20	TcCLB.511823.70, TcCLB.508323.9	Farnesyl pyrophosphate synthase, putative	OG4_11009	Yes	TcFPPS
IDI1	TcCLB.408799.19, TcCLB.510431.10	Isopentenyl-diphosphate delta-isomerase, putative	OG4_14499	Yes 6	TcIDI
ERG9	TcCLB.507897.20, TcCLB.508369.20	Farnesyl transferase, putative; squalene synthase, putative	OG4_13084	Yes	TcSQS
ERG1	TcCLB.509589.20, TcCLB.503999.10	Squalene monooxigenase, putative	OG4_13490	Yes	TcSQLE
ERG7	TcCLB.508175.70, TcCLB.506825.170	Lanosterol synthase, putative	OG4_11381	No	TcOSC
ERG11	TcCLB.506297.260, TcCLB.510101.50	Lanosterol 14-*α*-demethylase, putative	OG4_12975	Yes	Tc14DM, cCYP51
ERG24	TcCLB.507969.60, TcCLB.507129.30	C-14 sterol reductase, putative	OG4_12018	Yes	Tc14SR
–	TcCLB.511339.20, TcCLB.511895.69, TcCLB.509235.20	C-5 sterol desaturase, putative	OG4_20087	No	TcSMO-like
ERG26	TcCLB.510873.10	NAD(P)-dependent steroid dehydrogenase protein, putative	OG4_12533	No	TcNDSDHL
ERG27	–	–	–	No	–
ERG6	TcCLB.504191.10, TcCLB.505683.10, TcCLB.510185.10	Sterol 24-C-methyltransferase, putative	OG4_13307	Yes	Tc24SMT
ERG2	TcCLB.510329.90	C-8 sterol isomerase, putative	OG4_14573	Yes	Tc8SI
ERG3	TcCLB.473111.10, TcCLB.507853.10	Lathosterol oxidase, putative	OG4_12421	No	TcSC5D
ERG5	TcCLB.506945.190	Cytochrome p450-like protein, putative	OG4_14688	No	TcSC22D
ERG4	TcCLB.506577.120, TcCLB.507709.90	Sterol C-24 reductase, putative	OG4_16908	Yes	TcSC24R

The table lists *T. cruzi* genes mapped to either the corresponding KEGG maps, or the yeast SBPs (see Table 1). OrthoMCL identifiers are from the OrthoMCL Database version 4. *T. cruzi* gene identifiers are those currently available at the TriTrypDB resource. The fifth column shows whether the gene was mapped to the corresponding KEGG metabolic map at the time of this writing. The last column shows the nomenclature used in this work. Some of these gene names were previously used in the literature, and others are used here for the first time, based on the gene names of relevant orthologs. In the case of the TcIDI gene, the two listed alleles are truncated copies of the gene. The full-length gene is deposited in GenBank as AJ866772. While in the case of the TcNSDHL gene, the copy annotated by the genome project is truncated due to genome assembly problems. The full-length gene is deposited in GenBank as JN050853 (this work).

At this point, there were still two gaps present when modeling the *T. cruzi* SBP on top of the yeast pathway. These correspond to the enzymatic reactions catalyzed by the yeast genes ERG27 (3-keto sterol reductase) and ERG25 (C-4 methyl sterol oxidase). Our failure at identifying the orholog of the yeast ERG27 gene came as no surprise, as the enzymes performing C-3 ketoreduction in land plants and sterol synthesizing bacteria are still unknown [42], [43].

However, a number of putative homologs of ERG25 in *T. cruzi* were readily identified in sequence similarity searches. In this case, it was difficult to discern which of these were the true orthologs of the yeast ERG25 gene.

### Apparent loss of ERG25 homologs in *T. cruzi* and *T. brucei*

**Figure 1:**
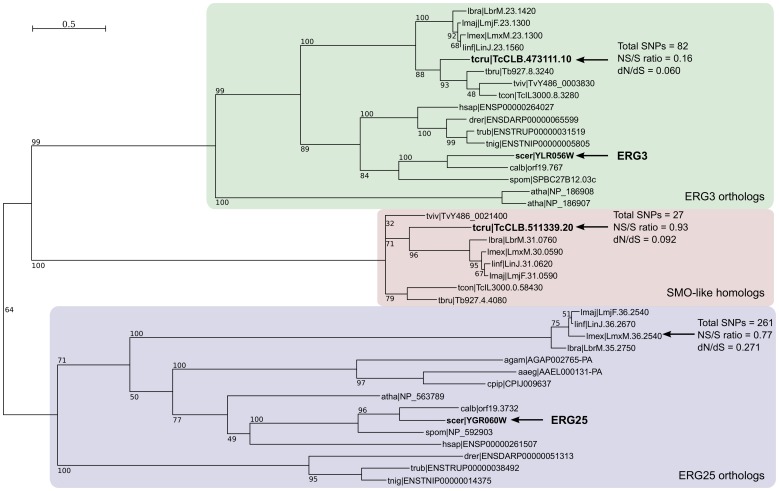
Phylogenetic tree of selected ERG3/ERG25 orthologs. Seleced orthologs from kinetoplastids, plants, vertebrates, invertebrates and fungi were aligned with t_coffee. A phylogenetic reconstruction was calculated using PhyML (LG model, bootstrap resampling with N = 1000). For clarity, highly similar genes/alleles were not included in the final tree. Organism abbreviations are: lbra  =  *L. braziliensis*; lmex  =  *L. mexicana*; linf  =  *L. infantum*; lmaj  =  *L. major*; tcru  =  *T. cruzi*; tviv  =  *T. vivax*; tcon  =  *T. congolense*; tbru  =  *T. brucei*; agam  =  *Anopheles gambiae*; aaeg  =  *Aedes aegypti*; cpip  =  *Culex pipiens*; hsap  =  *Homo sapiens*; calb  =  *Candida albicans*; scer  =  *S. cerevisiae*; spom  =  *Schizosaccharomyces pombe*; atha  =  *A. thaliana*; drer  =  *Danio rerio*; trub  =  *Takifugu rubripes*; tnig  =  *Tetraodon nigroviridis*.

A BLASTP search using the yeast ERG25p as query against kinetoplastid proteomes retrieved 5 *T. cruzi* significant hits, with 4 of them having a similar length (278-279 aa, See Table S1), sharing a common Pfam Domain (PF04116, Fatty Acid Hydroxylase Superfamily), and a common architecture with 3–4 predicted *trans*-membrane domains (the fifth gene is a truncated copy). These hits correspond to two pairs of alleles, and are currently annotated as 'C-5 sterol desaturases' in the *T. cruzi* genome database (or ‘lathosterol oxidases’, both are synonymous terms). As there is detectable similarity between ERG3 and ERG25 genes in yeast as well, a similar search using the yeast ERG3p as query retrieves the same set of *T. cruzi loci*. Therefore, to identify which *loci* correspond to the true orthologs of ERG25, we carried out a detailed phylogenetic analysis between these genes, in different organisms. Using the sequences of fungi, plant, insect, human, fish and trypanosomatid enzymes, we obtained a maximum-likelihood tree with a clear segregation of ERG25 and ERG3 orthologs in separate branches (Figure 1). The tree suggests that there is one *T. cruzi locus* (containing TcCLB.473111.10 and its TcIII-like allele, TcCLB.507853.10) that is the true ortholog of the yeast ERG3 gene. Interestingly, the branch containing ERG25 orthologs does not show genes from African or American trypanosomes (the only kinetoplastid genes are from Leishmanias). The other trypanosomatid genes identified in our BLAST searches are grouped together in a third branch, carrying only trypanosomatid genes. These results are consistent with reciprocal best-hits identified in BLAST searches (see Table S1). Reciprocal or bidirectional best hits provide support for the conjecture that the genes are equivalent orthologs [44], [45]. In these searches, there is a clear drop in the BLAST score when the query sequence is ERG3 or a ERG3 homolog. This score drop is not observed when the query sequence is ERG25 or an ERG25 homolog. Apart from this evidence, there is additional support for this separate branch from the BLAST searches, in the observed reciprocity of best hits. For example when using the *L. major* gene LmjF.36.2540 as query (grouped with ERG25 orthologs in Fig 1) the best hits are the yeast ERG25 and their orthologs. Also viceversa, when doing the reciprocal BLAST search using the yeast ERG25p as query, the best *L. major* hit is again LmjF.36.2540, even though in this case the scores are lower than for those obtained for ERG3 orthologs. These bidirectional hits are not observed for the group of trypanosomatid genes grouped in the middle branch in the phylogenetic tree.

Taken together, the most parsimonious interpretation of these results is that the C-4 sterol oxidase gene has been lost in the ancestor of *T. cruzi* and *T. brucei* (but not in the ancestor of Leishmanias). The presence of Leishmanial and Trypanosomal proteins grouped in the middle branch of the tree suggests that these group of homologs have a different ancestral origin. However, because C-4 demethylation is essential, we hypothesize that they may have a C-4 sterol oxidase activity (see Discussion). For these reasons we decided to call them Sterol Methyl Oxidase-like (SMO-like) in this work.

**Figure 2:**
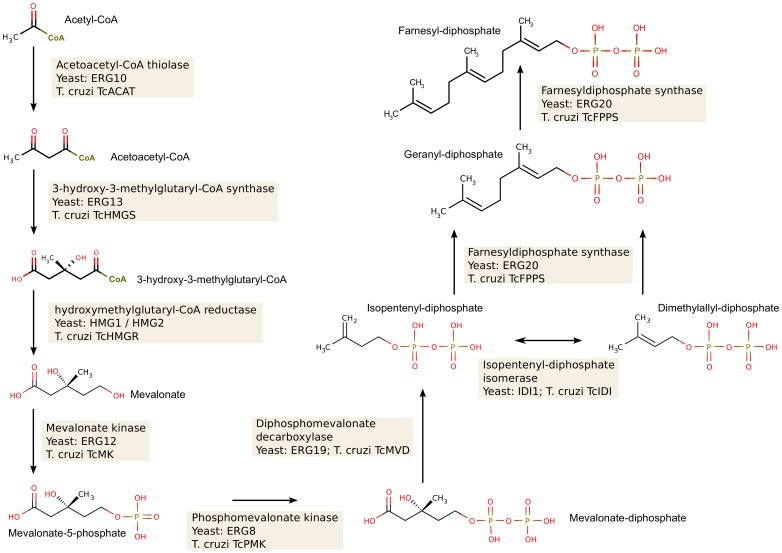
The *T. cruzi* isoprenoid biosynthesis pathway. The figure shows the metabolic steps leading to farnesyl-diphosphate, from acetyl-CoA, and the corresponding yeast and *T. cruzi* enzymes that catalyze these steps. Gene names used are those in Tables 1 and 2.

**Figure 3:**
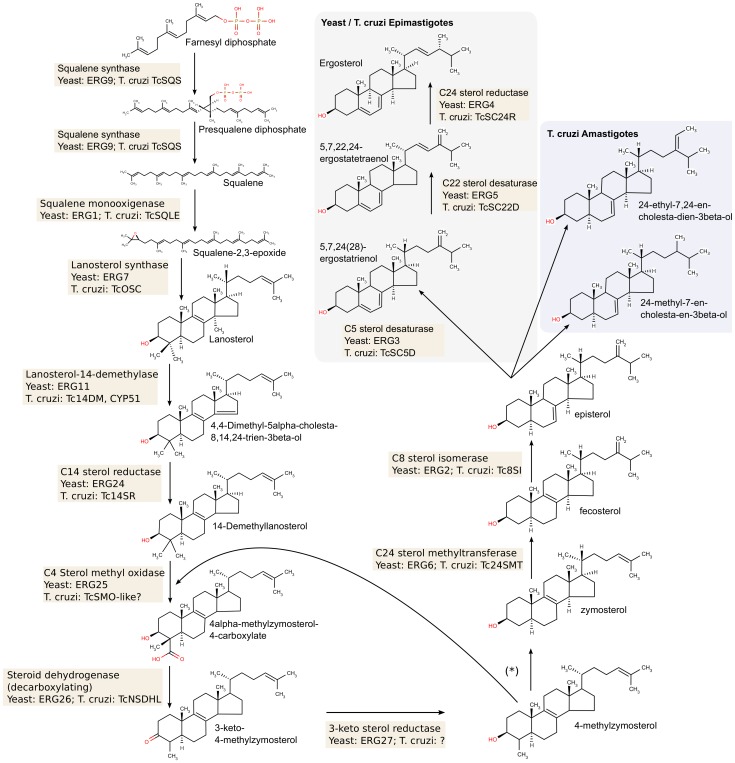
The *T. cruzi* sterol biosynthesis pathway. The figure shows the metabolic steps leading from farnesyl-diphosphate to ergosterol (in yeast, and in *T. cruzi* epimastigotes) or to different 24-alkylsterols (in *T. cruzi* amastigotes [64]), and the corresponding yeast and *T. cruzi* enzymes that catalyze these steps. The two methyl groups at C4 are removed in two rounds of successive C4-oxidation, C4-decarboxylation and C3-ketoreduction (*). Gene names used are those in Tables 1 and 2. Unknown/hypothetical assignments are shown with question marks.

Table 2 summarizes the results of our efforts to fill in the missing genes in the pathway, whereas Figures 2 and 3 show the reconstructed isoprenoid and sterol metabolic pathways, respectively. Once the *T. cruzi* SBP genes were identified, we proceeded to study the genetic diversity by re-sequencing these genes in a panel of *T. cruzi* strains, representative of all the extant evolutionary lineages of the parasite.

### Genetic diversity in the sterol biosynthesis pathway

To obtain sequence information from the selected genes we decided to use a methodology based on PCR amplification followed by direct sequencing. Therefore, it was important to reduce the possibility of amplification problems generated by polymorphisms that could prevent the annealing of the primers. A number of aspects were considered to reduce these risks i) we decided to focus our analysis on coding sequences where possible, as they are generally less variable than non-coding sequences; ii) we used the *T. cruzi* SNP database (TcSNP, http://snps.tcruzi.org) [46], to select the best regions for primer design, avoiding regions with candidate SNPs; and iii) we also limited the size of amplification products, to optimize the quality of the final sequence (see Methods). In this latter case, an optimal size was set to ∼750–800 bp, which would allow us to get complete sequence coverage, with good quality on both strands. Depending on the size of each gene, one or more overlapping amplification products had to be analyzed (the number of amplification products per gene is listed in Table 3).

**Table 3:** Comparative analysis of the quantity, density and type of SNPs identified in the sterol biosynthesis pathway of *Trypanosoma cruzi*.

	Polymorphisms (SNPs)						Length (bp)		
	Synonymous (S)		Non synonymous (NS)		Ratio	Total	CDS	Resequenced	
Genes	No. (%)	density	No. (%)	density	NS/S	No.	bp	bp (%)	PCR Fragments
TcACAT	35 (75.00)	3.27	11 (25.00)	1.09	0.33	48	1188	1100 (92.59)	2
TcHMGS	37 (86.05)	2.66	6 (13.95)	0.43	0.16	43	1497	1390 (92.85)	2
TcHMGR	27 (79.41)	2.18	7 (20.59)	0.56	0.26	34	1356	1240 (91.45)	2
TcMK	31 (79.49)	3.20	12 (30.77)	1.24	0.39	50*	1086	970 (89.32)	2
TcPMK	35 (53.85)	2.65	33 (50.77)	2.50	0.94	69*	1428	1320 (92.44)	2
TcMVD	32 (62.75)	3.08	19 (37.25)	1.83	0.59	51	1140	1040 (91.23)	2
TcIDI	25 (78.13)	2.50	7 (21.88)	0.70	0.28	32	1071	1000 (93.37)	2
TcFPPS	22 (66.67)	2.27	11 (33.33)	1.13	0.50	36*	1275	970 (76.08)	2
TcSQS	32 (69.57)	2.88	14 (30.43)	1.26	0.44	46	1212	1110 (91.58)	2
TcSQLE	50 (72.46)	3.07	21 (30.43)	1.29	0.42	73*	1716	1630 (94.99)	3
TcOSC	61 (66.30)	2.53	31 (33.70)	1.29	0.51	92	2706	2410 (89.06)	4
Tc14DM	47 (87.04)	3.46	7 (12.96)	0.51	0.15	54	1443	1360 (94.25)	2
Tc14SR	33 (63.46)	2.58	19 (36.54)	1.48	0.58	52	1371	1280 (93.36)	2
TcSMO-like	14 (51.85)	2.09	13 (48.15)	1.94	0.93	27	837	670 (80.05)	1
TcNSDHL	21 (70.00)	1.75	9 (30.00)	0.75	0.43	30	1221	1200 (98.28)	2
Tc24SMT	17 (77.27)	2.07	5 (22.73)	0.61	0.29	22	1050	820 (78.10)	1
Tc8SI	18 (78.26)	3.16	5 (21.74)	0.88	0.28	23	654	570 (87.16)	1
TcSC5D	70 (89.74)	9.72	11 (14.10)	1.53	0.16	82*	834	720 (86.33)	1
TcSC22D	29 (69.05)	2.09	13 (30.95)	0.94	0.45	42	1518	1390 (91.57)	2
TcSC24R	53 (75.71)	3.79	17 (24.29)	1.21	0.32	70	1467	1400 (95.43)	2
**Total/Avg**	692 (71.71)	2.93	273 (28.29)	1.16	0.39	975	26070	23590 (90.50)	39

SNP counts marked with * indicate totals that do not result from the sum of synonymous + non-synonymous substitutions, in some cases because of the presence of two substitutions in the same codon, and in other cases, because some SNPs fall in the non-coding region of the gene.

All amplification products were analyzed with a software package (PolyPhred, see Methods) that allows the identification of heterozygous peaks in the chromatograms. Therefore, for all selected genes, we identified two types of sequence polymorphisms: i) allelic variation within a strain/clone (heterozygous peaks identified by Polyphred); and ii) variation between strain/clones (identified in a multiple sequence alignment of sequenced products). For every gene, we identified the position of the variant sites, and the type of change introduced (synonymous or non synonymous). The complete information for each gene is available as supplementary material (Table S2), and a table summarizing these results is included herein (Table 3). Using this strategy, we generated ∼24 Kb of sequence, from 16 strains covering the 6 major *T. cruzi* evolutionary lineages. Overall we attained a coverage of ∼90% of the total coding sequence for the selected genes. From this analysis we identified 975 polymorphic sites, producing 965 codon changes, which generate 692 synonymous changes (72%) and 273 non-synonymous changes (28%, see Table 3). Interestingly, we uncovered heterozygous SNPs in all genes of the pathway, even for those which are currently represented in the reference genome in an haploid state (e.g. TcACAT, and TcSC22D). The genes Tc24SMT, TcHMGR and TcNSDHL had the lowest SNP density (2.68, 2.74 and 2.50 every 100 bp respectively), while TcSC5D had the highest SNP density (11.39 every 100 bp). This density is at least double that which is found in other SBP genes. Nevertheless, this gene is under an apparently high purifying selection, as suggested by its low dN/dS ratio (Table 4). Upon further investigation of this gene, we noticed a number of informative SNPs that could be exploited in a lineage typing assay. Therefore, we performed a separate re-sequencing experiment for this gene, in an expanded panel of strains, and developed two alternative typing assays based on this *locus* [47].

**Table 4:** Comparative analysis of the genetic diversity present in the sterol biosynthesis pathway of different kinetoplastids.

	dN/dS (*ω*)		
Yeast gene, Std symbol	Af Tryps	Leish	Tcr
ERG10/ACAT	–	–	**0.028**
ERG13/HMGS	0.057	0.048	0.054
HMG1/HMGR	0.065	0.052	0.057
ERG12/MK	0.077	0.076	0.077
ERG8/PMK	**0.110**†	**0.122**†	**0.122**†
ERG19/MVD	0.067	0.072	0.059
IDI1/IDI	0.051*	0.043*	0.048*
ERG20/FPPS	0.060	0.061	0.065
ERG9/FDFT, SQS	0.085	0.087	0.093
ERG1/SQLE	**0.115**†	**0.107**†	**0.115**†
ERG7/LSS, OSC	**0.109**†	**0.113**†	**0.113**†
ERG11/14DM, CYP51	0.042*	0.040*	0.039*
ERG24/14SR, TM7SF2	**0.142**†	**0.109**†	**0.110**†
ERG25/SMO/SC4MOL	–	**0.271**	–
–/SMO-like	0.080	0.082	0.092
ERG26/NSDHL	0.051*	0.057	0.058
ERG27/3KSR	–	–	–
ERG6/24SMT	0.065	0.062	0.070
ERG2/8SI	**0.115**†	0.100	0.097
ERG3/SC5D	0.069	0.059	0.060
ERG5/SC22D	–	0.028*	**0.107**†
ERG4/SC24R	–	0.097	0.070
Average	0.080	0.075	0.079
Standard deviation	0.028	0.028	0.026

The table shows the dN/dS (*ω*) ratio observed in the african trypanosome lineage (Af Tryps) (*T. brucei brucei*, *T. brucei gambiense*, *T. evansi*, *T. congolense*, *T. vivax*), in the *Leishmania* lineage (Leish) (*L. major*, *L. infantum*, *L. braziliensis*, and *L. mexicana*), and in the *T. cruzi* lineage (Tcr, sequences from TcI{TcVI DTUs). The values that are above (†) or under (*) a standard deviation from the average of each column are marked.

We next assessed the accumulation of changes at the protein level (missense polymorphisms), and observed that the genes TcSMO-like, TcMVD and TcPMK have the highest non-synonymous SNP density (1.82, 1.94 and 2.50 non-synonymous SNPs every 100 bp respectively). In spite of this, the dN/dS ratio for these genes is still low, ranging from 0.028 (TcACAT) to 0.122 (TcPMK), indicating the relatively high degree of conservation of *T. cruzi* SBP genes.

### Lineage-specific and intra-lineage differences

All the collected data on sequence diversity was also analyzed in the context of the current separation of *T. cruzi* in discrete evolutionary lineages. For this analysis we considered three types of polymorphisms or fixed differences: i) lineage specific polymorphisms (LSPs) (these are the polymorphic sites that could differentiate one lineage from all the others; ii) intra-lineage polymorphisms (ILP) (differences between strains belonging to the same lineage); and iii) heterozygous sites (those that differ between alleles of a single diploid individual). In this comparative analysis we found that the lineage TcI showed more than one LSP every 200 bp, while in the other lineages, the LSP density was at least four times lower (data not shown). This data agrees with the hypothesis that TcI is one of the ancestral lineages [6], [9]. Analyzing the ILP distribution, we found that the TcIV lineage presents the highest density of ILPs, with almost one ILP every 100 bp. Interestingly more than ∼90% of the ILPs observed in TcIV are restricted to the comparison between the South American strain CanIII with two strains from North America (Dog Theis and 92122102R, data not shown). The observed differences among TcIV strains from South and North America are in agreement with previous observations using different markers which suggested a phylogenetic divergence between TcIV from these two regions [4], [10], [48]–[54]. Indeed, our results show that the number of differences between CanIII and these other TcIV strains is even greater than those observed between lineage TcV and TcVI (the two hybrid lineages). Our results therefore agree with previous publications that describe a considerable phylogenetic distance between the CanIII and Dog Theis strains, even though they are still placed into the same evolutionary lineage based on current typing methods. As expected, the hybrid lineages (TcV and TcVI) show the highest level of heterozygosity, with more than 3 heterozygote sites every 200 bp; while TcII showed an intermediate level of heterozygosity with approximately 3 sites every 2 Kb, and the other lineages showed less than 10 sites in the 24 Kb analyzed.

### Potentially important non-synonymous changes

In many cases, variations in susceptibility or resistance to a drug are associated to mutations that occur near the interaction site of a substrate or an inhibitor. To analyze the potential relevance of the non-synonymous SNPs found, we gathered relevant information from the literature on the selected targets, their PFAM domains, PROSITE motifs, and the available crystallographic structures in the Protein Data Bank (PDB) for the *T. cruzi* enzyme or their orthologs (see Methods). Starting with these crystal structures, we first identified residues near the co-crystallized ligands (substrate, inhibitor or co-factor). For this we established a maximum distance limit of 7*Å* from each corresponding ligand. This analysis revealed a number of potentially important changes in the Tc14DM (TcCYP51), and TcMK genes.

**Figure 4:**
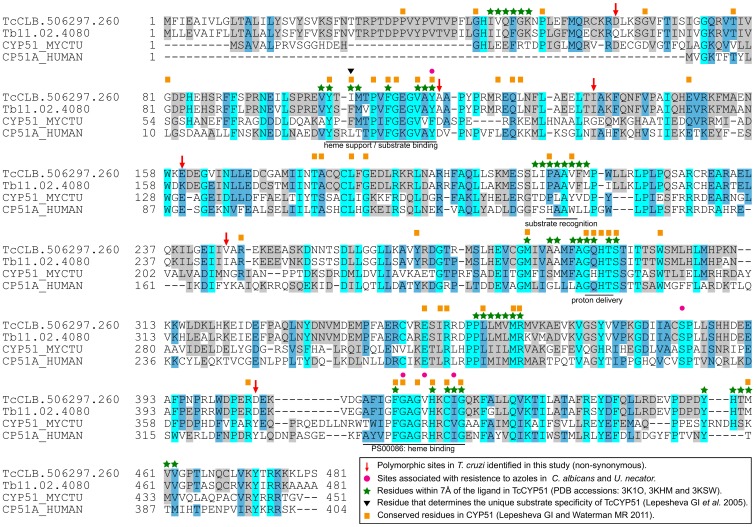
Alignment of *T. cruzi*, *T. brucei*, *M. tuberculosis* and human Lanosterol 14-*α* demethylases, showing the non-synonymous changes identified in this work (red arrows). Important residues either in Tc14DM or in the CYP51 family are noted [100], [101], as well as residues associated with resistance to azoles in *C. albicans* and *U. necator* [56], [57]. PS00086 is the Prosite Cytochrome 450 motif (cysteine heme-iron ligand signature).

In the azole target gene, Tc14DM (TcCYP51), we identified only 5 non-synonymous substitutions, none of which affect key residues of the enzyme (see Figure 4). However, after mapping these substitutions on top of the available TcCYP51 structure [55], we identified a number of potentially important substitutions that lie just next to important residues, or are within 7*Å* of the co-crystallized ligand. One of these is a A117S substitution that sits just next to a tyrosine (Y116) that is within 7*Å* of the ligand, and that has been shown to be involved in the generation of resistance to azoles in *C. albicans* and *U. necator* [56], [57]. In trypanosomatids, residue 117 marks the start of a short helix in the structure, that is uniquely found in trypanosomatids [58]. Alanine at residue 117 is present in *T. brucei* and was found in strains from the TcIII lineage in homozygosis and in heterozygosis in hybrid strains, while Serine at this position was found in homozygosis in strains from lineages TcI, TcII, and TcIV.

In the case of the TcMK gene, we identified 12 non-synonymous changes. One of these is a H29Y change, located in a conserved region of the sequence, close to a group of residues that are involved in substrate binding (at <7*Å* of the ligand (mevalonate) in the *L. major* structure (LmjF.31.0560, PDB: 2HFU) [59]. Histidine is encoded by the genes from strains M5631 and X109/2, both from lineage TcIII, while tyrosine is encoded in all other strains, including M6241, also of lineage TcIII. The second possibly important change in TcMK is a G287A substitution, also located in a very conserved motif of the MK gene, that is part of the GHMP kinase family domain (Pfam PF08544). This particular motif, contains a number of highly conserved glycines, some of which are within 7*Å* of the ligand in the *L. major* MK. In this polymorphic position strains from lineage TcV (Sc43 and Mn) encode glycine and alanine in heterozygosis, while all the other strains encode glycine in homozygosis.

For other targets, crystallographic structures were not available, and so we resorted to analyze non-synonymous substitutions in the context of functionally important motifs or domains. In the case of the squalene epoxidase gene (TcSQLE), we identified two changes (S306G and I307L) that stand between highly conserved proline residues (at 95% and 94% identity within the squalene epoxidase domain, PF08491). The I307L substitution is apparently conservative, as these are the two most frequent residues in this position in the Pfam domain. In contrast, the S306G substitution introduces a glycine that is completely absent at this position in the 429 sequences currently available for this protein family in the Pfam database. Serine is the second most frequent residue in this position, and is present in 14% of the sequences, all from fungi (glutamine is the most frequent residue, in 50% of the sequences). The Ser residue occurs in lineage TcII in homocygosis, and in lineages TcV and TcVI in heterocygosis, and its conservation in fungi suggests that this is the ancestral character at this position. Finally in the C-14 sterol reductase gene (Tc14SR), we identified two consecutive substitutions (P208H, V209F) in strains 92122102R and Dog Theis (lineage TcIV), that fall within the sterol reductase family signature motif (PS01017). However the substitutions are conservative, because they are both described by the motif pattern.

### The phosphomevalonate kinase of *T. cruzi* is not under a strong purifying selection

The phosphomevalonate kinases (PMKs) of pathogenic bacteria, fungi, and trypanosomes are attractive targets for the design of selective inhibitors, because the same phosphorylation reaction in humans and other animals is catalyzed by a non-orthologous enzyme [60]. In the first group of organisms, phosphorylation of mevalonate is performed by orthologs of the yeast ERG8 gene, while in animals it is performed by a group of orthologs of the human PMK gene (hPMK). These two groups of enzymes differ in a number of kinetic, biophysical properties, and in the ATP-binding motifs (ERG8-like kinases contain a protein kinase motif, while orthologs of the human enzyme have a P-loop or “Walker A” motif)[60], [61]. Analysis of the TcPMK gene in different strains of *T. cruzi* showed that the gene has accumulated 68 changes (35 synonymous, 33 non-synonymous, see Table 3), and 1 non-sense substitution (see below) in these independently evolving lineages. Based on the dN/dS ratio, the gene is under purifying selection (dN/dS < 1, see Table 4). However, it displays the highest dN/dS ratio of the pathway, the highest density of missense SNPs (2.5 non-synonymous SNPs/100 bp), as well as the highest ratio of synonymous to non-synonymous SNPs (∼1). Taken together, these data show that this is the gene that is apparently under more relaxed selection in the SBP pathway of *T. cruzi*.

Moreover, an interesting substitution was observed at codon 136 (407 bp) in this gene in the *T. cruzi* strain IVV (TcII). In this strain, one allele encoded a Serine (TCA), as in all other strains, whereas the second allele encoded a premature STOP codon (TAA). All ERG8-like PMKs are composed of two GHMP kinase domains: an N-terminal domain (Pfam PF00288) located in the middle of the protein (starting at residue 160 in *T. cruzi*), and a C-terminal domain (PF08544) closer to the C-termini. In *T. cruzi*, the nonsense mutation in the IVV strain is located upstream of this first domain (PF00288). Considering that the next possible translational start codon is located downstream of this domain, the IVV strain is therefore probably producing a very short non-functional protein from this allele, or two truncated proteins, both devoid of this domain. Thus, the most plausible hypothesis is that the IVV strain carries only one functional PMK allele, and can be considered naturally hemizygous for the PMK gene.

### Comparative analysis of genetic diversity in kinetoplastid sterol biosynthesis pathways

Although phylogenetically related, kinetoplastid parasites have evolved different adaptations to their host environments. This is particularly evident in the case of ergosterol dependency: both *T. cruzi* and *Leishmania* have an essential requirement for ergosterol and/or other 24-alkylsterols, and are unable to survive by salvaging cholesterol from the host [62]. Whereas sterol biosynthesis in *Trypanosoma brucei*, is apparently suppressed in bloodstream forms, relying instead on receptor-mediated endocytosis of host low-density lipoproteins carrying cholesterol [63]. Apart from differences in their strict requirement for *de novo* synthesis of sterols, there are also differences in the exact type and abundance of synthesized sterols, which may be explained by differences in the complement of genes and/or their activities or regulation. As an example, it has been described that *T. cruzi* amastigotes lack Δ^5,7^ sterols, suggesting a lack of Δ^5^ desaturase activity in this life-cycle stage [62], [64]. Based on these premises, we decided to investigate the accumulation of nucleotide changes (fixed differences) in genes from the sterol biosynthesis pathway in African Trypanosomes, and Leishmanias, reasoning that the selection acting on these genes could be different in each case. For this we identified the corresponding orthologs of the yeast and *T. cruzi* genes used in this work (see Table 2) in currently available kinetoplastid genomes (see Methods). The available information includes that of *T. brucei brucei* (2 strains), *T. brucei gambiense*, *Trypanosoma evansi*, *Trypanosoma congolense* and *Trypanosoma vivax*; and that of 4 subspecies of *Leishmania* (major, infantum, braziliensis and mexicana). Using this information we proceeded to analyze the nucleotide substitutions observed in each group. A summary of this analysis is presented in Table 4 (and in Table S3). Apart from the absence of some genes that were already discussed, other notable missing genes were the orthologs of the ERG10 (ACAT/thiolase) in African Trypanosomes and Leishmanias, and the ERG5 (SC22D) and ERG4 (SC24R) in *T. brucei*. The reaction catalyzed by the orthologs of ERG10 in other trypanosomatids, is catalyzed in *T. brucei* by another thiolase [15] (Tb927.8.2540, ortholog of POT1, see Table S4). However, whereas the absence of the sterol reductase has been noticed by others recently [65], we also noticed the absence of a gene encoding the C22 sterol desaturase in this organism.

Because the genetic distances within each phylogenetic group are different, it would be perhaps incorrect to compare nucleotide changes for each gene across these groups of organisms in a direct way. However, it is still possible to analyze and compare the information on sequence diversity within each group (e.g. column-wise in Table 4). In the table, we marked the genes with the highest and lowest dN/dS ratios within the pathway, and those deviating > 1 standard deviation. When looking at data in this way, a number of observations can be made: the less and most diverse genes in each pathway correlate very well: PMK, SQLE, OSC and 14SR belong to the most diverse genes of the pathway, while IDI1 and 14DM are the most conserved genes in all species. The only exceptions are the NSDHL and 8SI, which belong, respectively, to the less and most conserved genes only in African trypanosomes, and SC22D, which belongs to the group of highly conserved genes in Leishmania but to the group of less conserved genes in *T. cruzi*. Altogether, these data show that although there is a general high apparent purifying selection over the SBP of trypanosomatids (all dN/dS values are <<1), there are differences in the restrictions imposed to diversification in each group of organisms.

## Discussion

The main goal of our work was to study the genetic diversity present in the isoprenoid and sterol biosynthesis pathway (SBP) of *T. cruzi*. These pathways are validated chemotherapeutic targets for Chagas Disease, with a number of compounds currently undergoing clinical trials. Because the *T. cruzi* SBP pathway appeared to be incomplete in metabolic pathway databases such as KEGG when we started this work, and because the annotation of the SBP genes was also incomplete, we had to perform a small-scale bioinformatics analysis to fill in the gaps in available sequence and annotation. This task was performed primarily based on the well studied *S. cerevisiae* ergosterol biosynthesis pathway. As a result of this strategy, the majority of the genes of the pathway have now been identified, with the exception of the orthologs of the yeast gene ERG27, which encodes a 3-keto sterol reductase. As mentioned, the gene(s) responsible for this activity in land plants and sterol synthesizing bacteria have not been identified yet [43]. It is therefore highly likely that the trypanosomatid 3-keto sterol reductase is phylogenetically closer to the plant enzymes, and that once this elusive gene is identified it will be readily identified in trypanosomatids.

We selected 21 genes from this pathway to build a genetic diversity profile from representative strains of the six major evolutionary lineages of *T. cruzi*. For this analysis we used at least 2 strains for each evolutionary lineage therefore effectively sampling a large genetic space. Although it is certainly likely that other SNPs or fixed differences can be discovered when sequencing from new isolates, most probably these new mutations will correspond to changes that are unique to the new isolate (e.g. introduced during the clonal expansion of this particular isolate). In our experience, when expanding our re-sequencing analysis of the TcSC5D gene with 13 additional strains from lineages TcI-TcVI, only 6 new polymorphic sites were identified (see Fig S1 in [47]). Considering that this is the gene with the highest density of SNPs in the panel (82 polymorphic sites found in the present analysis), by doubling the number of strains sampled we only obtained a mild increase of sequence diversity information. Thus, we consider that in the current study we have covered a significant amount of the genetic diversity space of these pathways.

The strategy employed consisted in the generation of overlapping amplification products of approximately 750 bp (with ∼100 bp of overlap) for each gene, followed by direct sequencing of amplification products in both strands. As a result, the majority of the sequenced bases were read at least twice, with coverage in both strands. The primers were designed based on the CL-Brener genome sequence, and the majority of them were designed against the corresponding coding sequence to reduce the possibility of amplification problems (under the hypothesis that coding sequences are much less polymorphic than non-coding sequences). Moreover, when designing primers we avoided SNPs already identified from sequences in the public domain by checking against the TcSNP database. This strategy enabled the amplification and sequencing of all the selected gene fragments in strains from all the lineages, except for the first amplification product of the TcMK gene, that could not be amplified initially from lineage I. These strains carry a SNP that is specific for TcI and that mapped exactly at the 3'-end of the forward primer (see Table S5). We fixed this primer after recent genomic data was made available for a number of Tcruzi I strains (Sylvio X10 [66], JR cl4 (accessed trough TriTrypDB [67]), and TcAdriana (Westergaard G and Vazquez M, unpublished results).

Our results show that the TcSC5D gene is the most polymorphic gene in the pathway, with 11 SNPs per 100 bp (for a total of 82 SNPs in 720 bp analyzed). This SNP density is more than twofold larger than those observed for other re-sequenced genes. Despite this, the gene is under strong purification pressure, as judged by the ratio of missense and sense SNPs (or its dN/dS ratio). The Tc14DM and TcIDI1 genes had the lowest dN/dS ratios in the panel. Interestingly, the orthologs in Leishmanias and African trypanosomes also display the lowest ratios in each case (See Table 4), highlighting the level of apparent selection pressure for these enzymes in trypanosomatids. Furthermore the Tc14DM, TcHMGS and TcHMGR genes have the lowest density of missense SNPs in the panel, with only 0.51, 0.43 and 0.56 non synonymous SNPs every 100 bp respectively, so at least from these genetic evidence alone, these genes would appear to be the best candidates for drug development in *T. cruzi*.

### The SMO-like (or SC5D-like) genes of *T. cruzi* and Leishmania

As described above, when looking for the *T. cruzi* orthologs of the ERG25 (C4-methyl oxidase) gene, we identified two homologs (two pairs of allelic variants from the hybrid genome), which are members of the Fatty Acid Hydroxylase superfamily (Pfam Domain PF04116). Based on best-reciprocal BLAST hits and careful examination we concluded that these genes (TcCLB.511339.20, TcCLB.509235.20) are not orthologous to ERG25, and are probably divergent homologs of a different ancestral gene. Our phylogenetic reconstruction suggests that Leishmanias have retained the ancestral ortholog of ERG25, although the apparent selection acting on this gene is more relaxed than that observed for other genes in the pathway (see Table 4). In any case, based on this analysis it is tempting to speculate that the group of genes we call SMO-like, which are present in all trypanosomatid species analyzed in this work, are the ones that are responsible for the essential C4-demethylation step.

Interestingly, when performing BLASTP searches of SMO-like proteins against fungal genomes, we noticed a number of SUR2 (Sphinganine C4-hydroxylase) homologs among the significant hits (see Table S1). In yeast, SUR2 (also a member of the FA hydroxylase superfamily) catalyzes the conversion of sphinganine to phytosphingosine in sphingolipid biosynthesis. Recently, the presence of phytosphingosine in trypanosomes was demonstrated by mass spectrometry [68], and the authors also show that the biosynthesis of phytosphingosine is driven by bi-functional hydroxylase/desaturase enzymes. However, the trypanosomatid genes they identified as responsible for this activity are not orthologs of yeast SUR2 (see Figure 2 in Vacchina *et al.* [68]). In our reciprocal BLAST searches using the yeast SUR2 protein as query against trypanosomatid genomes, we always retrieve the same set of ERG3 orthologs, and SMO-like genes. Overall these data suggest that in trypanosomes the orthologs of both SUR2 and ERG25 have been lost, and that the SMO-like genes grouped in the middle branch in Figure 1 could represent an ancestral hydroxylase/desaturase that has adjusted (or is still adjusting) to a new functional niche in these organisms (cellular localization or time/stage of expression, etc). Interestingly the expression of this gene is higher in amastigotes and trypomastigotes, the two life cycle stages that occur in the mammalian host (data from Minning *et al*. [69]).

The protein sequences encoded by these genes show the three canonical conserved histidine boxes (HxxxH, HxxHH, and HxxHH) present in all fatty acid hydroxylase family members. The distribution of the accumulated changes is shown in Figure S1. None of the non-synonymous changes affect these highly conserved motifs, and at least a third of these (depending on the membrane topology prediction) are predicted to be exposed (not embedded in the membrane). This is important because, as reviewed in [70], the evolution of new regioselectivities in these enzymes would not involve the active site, but adjacent sequences. However, the failure to predict a reasonable topology (see Fig S1) points to the need to do an in-depth study of the membrane topology of this protein.

### The use of dN/dS ratios

Although appealing because it normalizes substitutions over silent vs non-silent sites, the dN/dS ratio is extremely sensitive to violation of a number of assumptions, such as the need to use data from divergent lineages, and is also affected by differences in the time since divergence and/or effective population sizes of compared species, among others [71]. This has been noticed in many studies, particularly in the case of bacterial species (even those with highly clonal populations) [72]. As shown by these and other authors, the dN/dS ratio is sometimes unstable, displaying a time dependency that makes it difficult to compare genomes at differing levels of divergence [73].

In preliminary studies with genes from these pathways, we came across a number of puzzling observations: some highly conserved genes in *T. brucei* showed unusually high (>>1) dN/dS values. This was due to the restricted number of genomes analyzed (3), which resulted in a low SNP frequency. Therefore the dN/dS ratio calculation did not provide enough depth for a reliable analysis as most genes had very few (or none) synonymous substitutions. This effect disappeared when adding sequences from other african trypanosomes (more distant lineages), which prompted us to investigate dN/dS trajectories.

As shown in the Figure S2, for pairwise comparisons where the number of polymorphisms is low, the dN/dS ratios are unstable and tend to display large values particularly for comparisons between highly conserved pairs of sequences. This should not be interpreted as an apparent diversifying selection, as these dN/dS values tend to (paradoxically) stabilize when more divergent sequences are compared. These results are in line with previous observations, and point to the use of care when analyzing dN/dS values. As shown by others [71]–[73] the use of dN/dS should be restricted to sequences from divergent lineages (with no sexual exchange among them). However, in the case of trypanosomatids this is difficult to enforce. Both within the *T. cruzi* and *Leishmania* lineages there are natural hybrids [7], [8], [49], [74], [75], which suggests that, although infrequent, genetic exchange have occurred in the wild. Therefore, the possibility of recombination between the parental alleles in these hybrids cannot be excluded. This is also the case for *T. brucei*, which has an extant capacity for both clonal and sexual propagation with varying degrees of inbreeding or out-crossing, with *T. brucei gambiense* showing a strict clonality [76], whereas different subpopulations of *T. b. rhodesiense* show both clonality and epidemic or close to panmictic behavior [77].

For all these reasons we believe that extreme care should be used when interpreting the meaning of isolated dN/dS values (e.g. in Table 4), or the meaning of comparisons of dN/dS across taxa in the absence of the expected trajectories of dN/dS over time (because of the critical effect of time since divergence of the lineages). This makes it difficult to answer simple questions such as whether the C-3 sterol dehydrogenase gene (ERG26 ortholog) is under the same apparent selection in *T. cruzi*, the African trypanosomes and Leishmanias. The Table 4 shows that the dN/dS values for this gene are similar in all cases (0.058 in T. cruzi, 0.057 in Leishmania, and 0.051 in African tryps). However, these genes differ wildly in their SNP densities (see Table S3), which makes it difficult to provide a simple answer.

## Conclusions

In this study we have explored the genetic diversity present in an important pathway of *T. cruzi*, which is the current target of a number of drugs undergoing clinical trials. Our analysis show that the targets of current drugs are highly conserved across all evolutionary lineages. We have also filled a number of holes in the pathway by completing the sequences of a number of genes that were missing or truncated in the current reference genome. Finally by comparing genes across other important trypanosomatids, we show than in spite of differences in diversity, all trypanosomes show a mostly conserved set of enzymes.

## Materials and Methods

### *T. cruzi* stocks and strains

Strains used in this study (and the corresponding current lineage classification) were: Sylvio X10 cl1 and Dm28 (TcI); MAS1 cl1, TU18 cl93, IVV cl4 (TcII); M6241 cl6, M5631 cl5 and X109/2 (TcIII); CanIII cl1, Dog Theis and 92122102R (TcIV); Sc43 cl9 and MN cl2 (TcV); Tulahuen cl2, CL Brener and P63 cl1 (TcVI).

### Oligonucleotides and gene identifiers

For each selected gene, a number of primers were designed for PCR-amplification. Using information from the TcSNP resource, we selected primers to avoid known polymorphic bases. Taking into account that in the direct Sanger sequencing of PCR products the chromatogram quality is optimal in the range from 50 to 700 bp, a desirable length of the amplification products was set around 750 bp. This length would also maximize our ability to sequence both strands of the amplification product, with good quality. Depending on the size of each selected gene, one or more overlapping amplification products were obtained. The list of the designed primers for each gene and the size of the corresponding amplification product is shown in Table S5. In only one case we had to design a separate primer (Tc-Mev-kinase26-TcI-fw) to amplify a fragment in one lineage (TcI) because there was a SNP at the 3' end of the primer that was absent in the release 1 of the TcSNP database.

### Amplification and sequencing

Selected fragments were amplified by PCR using Taq polymerase (Invitrogen) in a Biometra T Professional Gradient 96 cycler. Amplification mixtures contained 10 pmol of each primer, PCR buffer (Invitrogen), 1.5 mM MgCl^2^, 50 ng of genomic DNA, 200 µM dNTPs, 2.5 U Taq polymerase (Invitrogen), and water to a final volume of 25 µl. After denaturing at 94°C for 2 minutes, thermal cycling was performed for 35 cycles at 94°C for 30 seconds, followed by 30 seconds at a temperature set to 5°C less than the melting temperature of the selected primers, followed by 72°C for 30 seconds. Reactions were finished by a 5 minute incubation at 72°C. Amplification products were checked in 1.2% agarose gels stained with ethidium bromide to verify the presence of a single amplification product. Next, an aliquot (10 µl) of the amplification reaction was treated with 1 U of Exonuclease I (Fermentas) and 10 U of Shrimp Alkaline Phosphatase (Fermentas) for 45 minutes at 37°C and then for 30 minutes at 80°C to inactivate these enzymes. Subsequently two sequencing reactions were prepared, each with one of the primers used for the amplification of the product. Sequencing was carried out in an Applied Biosystems 3130 capillary sequencer using a Big-Dye terminator cycle sequencing kit, according to the instructions of the manufacturer.

### SNP identification and scoring

Gene fragments were PCR-amplified from every strain of the panel and sequenced in both strands. Base calling of chromatograms, assembly of sequences, detection of polymorphisms and manual inspection of assembled sequences and polymorphisms was done using a software package composed of Phred (version 0.020425.c) [78], [79], Phrap (version 0.9909329), Polyphred (version 5.04) [80] and Consed (version 15.0) [81]. Basecalling of chromatograms was done by Phred. Sequences were then assembled by Phrap. Polyphred was used to process phrap assemblies to detect polymorphic sites. All candidate SNPs identified by PolyPhred (score >70/99), including heterozygous peaks, were visualized with Consed. A few false positives, and false negatives were removed/added after this manual inspection.

### Other sequences used in this study

For the comparative analysis of SBP genes in kinetoplastid genomes, we have obtained the corresponding protein sequences for each of the yeast and/or *T. cruzi* orthologs used in this study from the TriTrypDB database [67]. A BLAST search using the corresponding SBP gene as query was used to identify the corresponding ortholog. This has been cross-checked by inspection of ortholog clusters at the OrthoMCL database [41]. The sequences used belong to the following species/strains: *T. brucei brucei* strains TREU927/4 [82] and Lister 427 (George Cross, unpublished), *Trypanosoma brucei gambiense* [83]; *Trypanosoma congolense* and *Trypanosoma vivax* [84]; *Trypanosoma evansi* (STIB 805) (unpublished); *L. major* [85], *L. infantum* [86], *L. braziliensis* [86], and *L. mexicana* [87].

### Alignment and calculation of dN/dS

For calculation of dN/dS ratios (*ω*) nucleotide sequences were first trimmed so that they all start and end at the corresponding START and STOP codons, and/or they have a similar length that is also a multiple of 3. Nucleotide alignments for each gene were then obtained using TranslatorX, which produces a codon alignment guided by aminoacid information [88]. Using these alignments as input, together with an unrooted phylogenetic tree with 3 branches containing *T. cruzi* lineages, Leishmania species and African trypanosome species, we ran the codeml program from the PAML package [89] with parameters “model = 0; NSSites = 0” to obtain optimized lengths for these branches by maximum likelihood. Finally, using these optimized branch lengths, we calculated the dN/dS ratio (*ω*) for each of the 3 branches by running again codeml with the following parameters:“model = 2; NSSites = 0”.

### Mapping substitutions on three dimensional structures

Crystallographic structures of enzymes of the sterol biosynthetic pathway were obtained from the Protein Data Bank (PDB, http://www.rcsb.org). Using the molecular graphics viewer VMD (version 2.8.6) [90] together with the multiple sequence alignment plugin for VMD [91] we mapped sequences from different strains on top of the reference sequence from which the structure was obtained. Atomic distances from each residue to the co-crystallized ligand were measured using standard tools implemented in VMD. Structures used in this work (and their source organism, and *T. cruzi* homolog) were: 1wl5 (human, TcACAT) [92]; 2fa3 (*Brassica juncea*, TcHMGS) [93]; 3bgl (human, TcHMGR) [94]; 2hfu (*L. major*, TcMK) [59]; 2hke (*T. brucei*, TcMVD)[95]; 1yhm (*T. cruzi*, TcFPPS) [96]; 1ezf (human, TcSQS) [97]; 1w6k (human, TcOSC) [98]; 3khm, 3k1o, 3ksw (*T. cruzi*, TcCYP51) [99].

### Data deposition

The sequences reported in this paper have been deposited in the GenBank database under the following accession numbers: JN050313-JN050853, HQ586972-HQ586973, and KF290395-KF290460. Heterozygous sequence polymorphisms have been submitted as ambiguities in the sequences using standard IUPAC notation. Sequence polymorphisms identified between different strains/clones are available as supplementary material, and will be also available in a future release of the TcSNP database (http://snps.tcruzi.org, [46]).

## Supporting Information

Figure S1 **Distribution of observed SNPs in the TcSMO-like genes of** ***T. cruzi*** **.** Based on the prediction of trans-membrane spanning domains (see TMHMM probability plot at the bottom), we created two alternative representations, following [70]. The distribution of synonymous and non-synonymous SNPs is shown according to these models. The representations differ in the presence/absence of the second (non-predicted) trans-membrane domain. In these two representations the location of the 3rd histidine box always lies on the opposite side of the membrane. Both topologies may be wrong and an in-depth study may be required to establish the correct topology of these proteins.
(PDF)

Figure S2 **Stability of the dN/dS ratio for the trypanosomatid isoprenoid and ergosterol biosynthesis genes.** The dN/dS ratio was calculated for all possible pairwise comparisons of each gene within each phylogenetic group/branch (e.g. the genes for all lanosterol demethylases were aligned with each other within the *T. cruzi* group, the Leishmania group and the *T. brucei*/African trypanosomes group). The plot therefore summarizes data from all genes in the pathways, and for all species analyzed. The dN/dS values were then sorted, grouped in bins of 10 and plotted. The error bars show the standard deviation within each bin.
(PDF)

Table S1 **Reciprocal BLASTP searches between fungal and kinetoplastid ERG25/ERG3 homologs.** The file contains a summary of BLASTP searches against fungal and kinetoplastid protein databases. BLAST searches using the yeast ERG3/ERG25 protein sequences as query, were run at the TriTrypDB BLAST server against a database of Kinetoplastid proteomes from reference and draft genomes. BLAST searches using a number of putative *T. cruzi* orthologs of these yeast genes were run at the SGD Fungal BLAST Server, against a database containing a selection of fungal genomes. Each BLASTP search is shown in a separate tab in the Excel workbook.
(XLS)

Table S2 **List of nucleotide changes (SNPs, fixed differences) identified for each gene analyzed.** The excel file contains one spreadsheet per gene with information on the location of each SNP relative to the start codon, the PolyPhred score for the SNP, and the character state of the SNP in each strain/lineage.
(XLS)

Table S3 **Comparative genetic diversity of kinetoplastid SBP genes.**
(XLS)

Table S4 **Presence of two types of thiolases in** ***T. cruzi*** **.** The file contains the summary of results from reciprocal BLASTP searches between fungal and kinetoplastid ERG10/POT1 homologs.
(XLS)

Table S5 **List of oligonucleotide primers and amplification products analyzed in this study.** The table lists the oligonucleotide primers used to amplify each PCR product, the corresponding product size, and the number of products per gene/locus.
(XLS)
