# Supplementary material for: Genetic Profiling of the Isoprenoid and Sterol Biosynthesis Pathway Genes of Trypanosoma cruzi
Source: PLoS One. 2014 May 14;9(5):e96762. doi: 10.1371/journal.pone.0096762 (PMC4020770; doi:10.1371/journal.pone.0096762)
Supplement: Figure S1 — Distribution of observed SNPs in the TcSMO-like genes of T. cruzi . Based on the prediction of trans-membrane spanning domains (see TMHMM probability plot at the bottom), we created two alternative representations, following [70]. The distribution of synonymous and non-synonymous SNPs is shown according to these models. The representations differ in the presence/absence of the second (non-predicted) trans-membrane domain. In these two representations the location of the 3rd histidine box always lies on the opposite side of the membrane. Both topologies may be wrong and an in-depth study may be required to establish the correct topology of these proteins. (PDF) [file pone.0096762.s001.pdf]

Figure S1. Cosentino RO and Agüero F. Genetic profiling of the isoprenoid and sterol biosynthesis pathways of Trypanosoma cruzi.

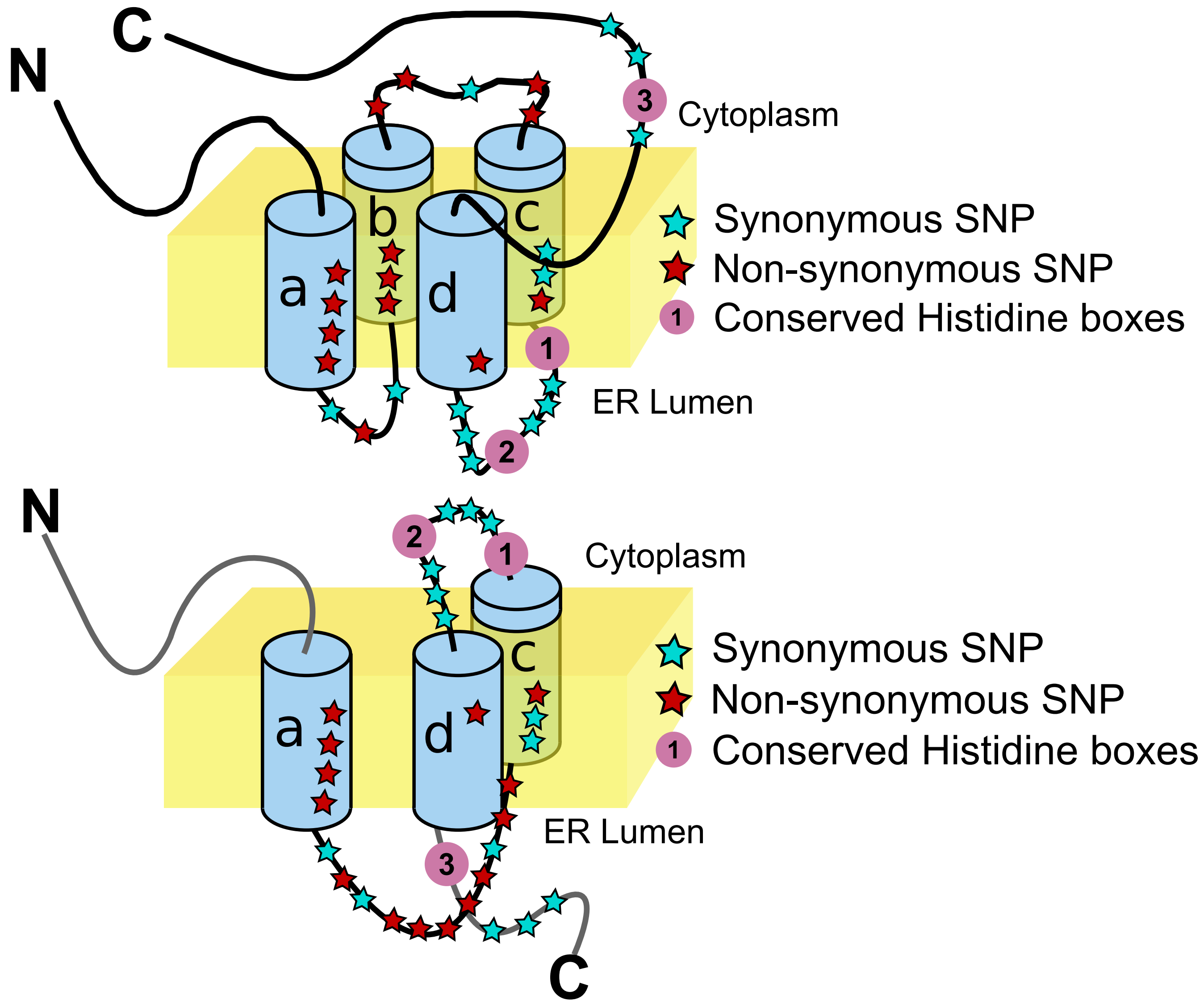

TcCLB.509235.20
TcCLB.511339.20
Sterol Methyl Oxidase - like

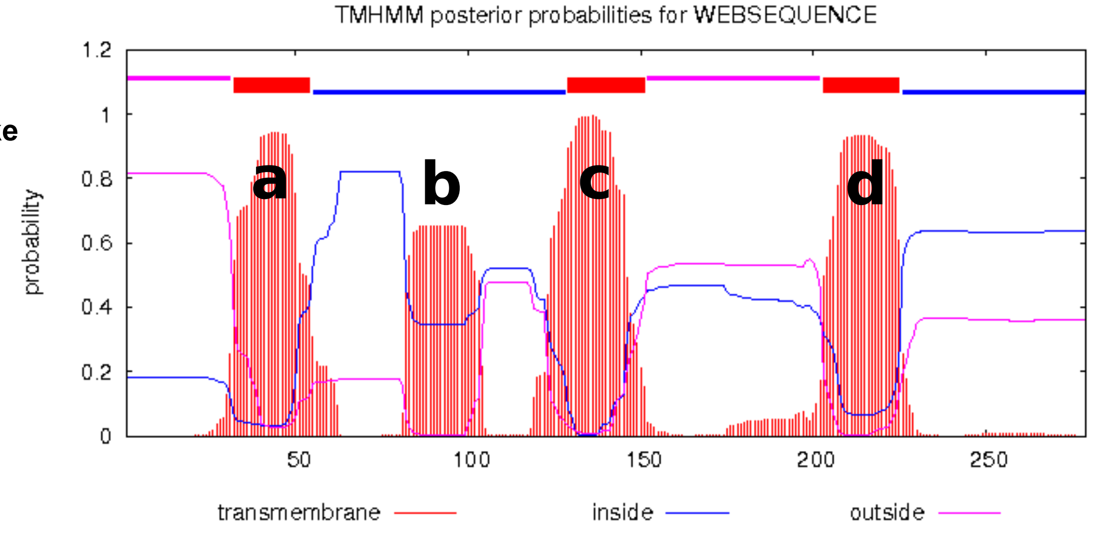

Distribution of observed SNPs in the TcSMO-like genes of T. cruzi. Based on the prediction of trans-membrane spanning domains (see TMHMM probability plot at the bottom), we created two alternative representations, following Sperling, Ternes, Zank, and Heinz (2003). The distribution of synonymous and non-synonymous SNPs is shown according to these models. The representations differ in the presence/absence of the second (non-predicted) trans-membrane domain. In these two representations the location of the 3rd histidine box always lies on the opposite side of the membrane. Both topologies may be wrong and an in-depth study (similar to the one performed by Diaz AR, et al, 2002, J Biol Chem 277: 48099) may be required to establish the correct topology of these proteins.
