# Supplementary material for: Genetic Profiling of the Isoprenoid and Sterol Biosynthesis Pathway Genes of Trypanosoma cruzi
Source: PLoS One. 2014 May 14;9(5):e96762. doi: 10.1371/journal.pone.0096762 (PMC4020770; doi:10.1371/journal.pone.0096762)
Supplement: Figure S2 — Stability of the dN/dS ratio for the trypanosomatid isoprenoid and ergosterol biosynthesis genes. The dN/dS ratio was calculated for all possible pairwise comparisons of each gene within each phylogenetic group/branch (e.g. the genes for all lanosterol demethylases were aligned with each other within the T. cruzi group, the Leishmania group and the T. brucei/African trypanosomes group). The plot therefore summarizes data from all genes in the pathways, and for all species analyzed. The dN/dS values were then sorted, grouped in bins of 10 and plotted. The error bars show the standard deviation within each bin. (PDF) [file pone.0096762.s002.pdf]

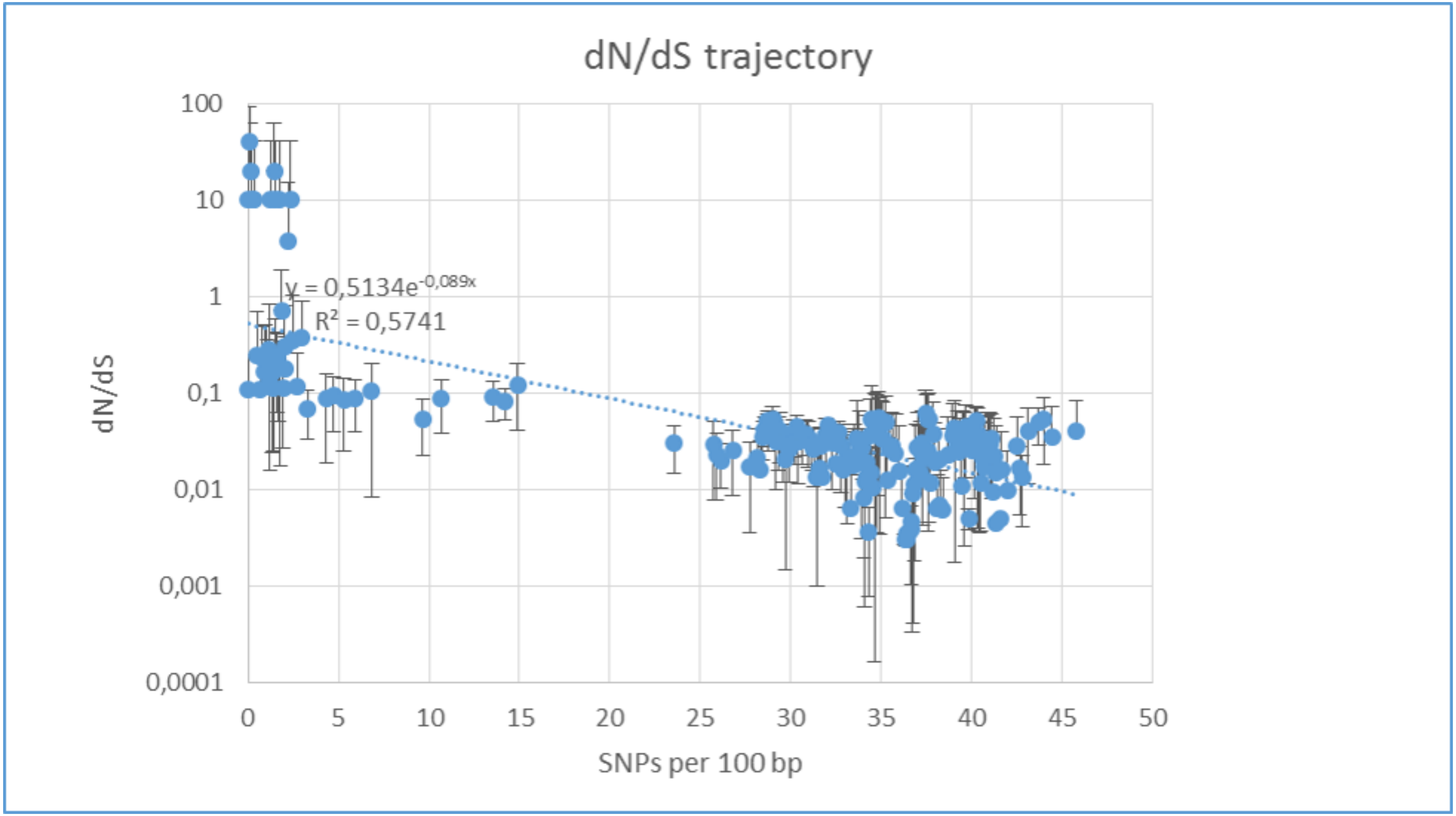

Stability of the dN/dS ratio for the trypanosomatid isoprenoid and ergosterol biosynthesis genes. The dN/dS ratio was calculated for all possible pairwise comparisons of each gene within each phylogenetic group/branch (e.g. the genes for all lanosterol demethylases were aligned with each other within the T. cruzi group, the Leishmania group and the T. brucei/African trypanosomes group). The plot therefore summarizes data from all genes in the pathways, and for all species analyzed. The dN/dS values were then sorted, grouped in bins of 10 and plotted. The error bars show the standard deviation within each bin.
